# Sensory Stimulation of the Triceps Surae Muscle Complex Modulates Spinal Reflex Responses—A Comparison between Tapotement Massage and Repetitive Peripheral Magnetic Stimulation (rPMS)

**DOI:** 10.3390/brainsci14020119

**Published:** 2024-01-24

**Authors:** Volker R. Zschorlich, Fengxue Qi, Jörg Schorer, Dirk Büsch

**Affiliations:** 1 Institute of Sports Science, Faculty of Philosophy, University of Rostock, Ulmenstr. 69-House 2, 18057 Rostock, Germany; 2 Institute of Sport Science, School IV—School of Humanities and Social Sciences, Carl von Ossietzky Universität Oldenburg, Ammerländer Heerstraße 114-118, 26129 Oldenburg, Germanydirk.buesch@uni-oldenburg.de (D.B.); 3 Department Aging of Individuals and Society, Faculty of Interdisciplinary Research, University of Rostock, Gehlsheimer Str. 20, 18051 Rostock, Germany; 4 Sports, Exercise and Brain Sciences Laboratory, Beijing Sport University, Beijing 100084, China

**Keywords:** sensory afferents, tapotement massage, muscle stiffness, motoneuron, tendon reflex, spasticity, neurorehabilitation, cerebral palsy

## Abstract

Background: The reduction of muscular hypertonia is important in the treatment of various diseases or rehabilitation. This study aims to test the efficacy of a 5 Hz mechanical muscle stimulation (tapotement massage) in comparison to a 5 Hz repetitive peripheral magnetic stimulation (rPMS) on the neuromuscular reflex response. Methods: In a randomized control trial, 15 healthy volunteers were administered with either 5 Hz rPMS, tapotement massage, or rPMS sham stimulation. The posterior tibial nerve was stimulated with rPMS and sham stimulation. The Achilles tendon was exposed to a mechanically applied high-amplitude 5 Hz repetitive tendon tapotement massage (rTTM). The tendon reflex (TR) was measured for the spinal response of the soleus muscle. Results: After rPMS, there was a reduction of the TR response (−9.8%, *p* ≤ 0.034) with no significant changes after sham stimulation. Likewise, TR decreased significantly (−17.4%, *p* ≤ 0.002) after Achilles tendon tapotement intervention. Conclusions: These findings support the hypothesis that both afferent 5 Hz sensory stimulations contributed to a modulation within the spinal and/or supraspinal circuits, which resulted in a reduction of the spinal reflex excitability. The effects could be beneficial for patients with muscle hypertonia and could improve the functional results of rehabilitation programs.

## 1. Introduction

In neurorehabilitation, patients often appear with various forms of movement disorders. They frequently suffer from spasticity, especially when they have lesions in their upper motor neurons or issues such as damage to the spinal cord. Numerous functional concerns, such as elevated muscle tone, heightened tendon jerks [1], and functional movement restrictions, can result from spasticity [2]. Spastic symptoms are characterized by an increase in tonic stretch reflexes that increases with velocity [3]. Treating muscle hypertonia is extensively used in rehabilitation practice since this hypertonia causes problems like discomfort or painful muscle tension and restrictions in musculoskeletal function. Great efforts are being made to treat these types of motor disorders, and attempts are being made to test new methods for their effectiveness in reducing muscle hypertension.

Common treatment methods include various forms of skeletal muscle massage [4,5,6]. The aim of massage in physiotherapy practice is to have a beneficial impact on muscle hypertonia and muscle spasticity and improve motor proficiency. A review of the neurophysiological and biomechanical effects of massage found inconsistent results across studies. The different forms of massage, the length of time they are applied, and a lack of placebo-controlled group-designed studies could be contributing factors to this variability [7]. In an early study of massage with two different intensities (light and deep kneading for 3 min), the inhibitory effect was only very short-term [8]. After the massage was stopped, the inhibition of the H-reflex amplitude returned to baseline within ten seconds. A reduction of 25% in the H-reflex amplitude was recorded in another experiment by the same group [9]. Applying a focal vibration treatment to the Achilles tendon every day for 14 days (50 Hz, 0.1 mm amplitude, 1 h) produced a significant decrease in the TR amplitude. Additionally, there was a significant reduction in the biomechanical stiffness of the muscle-tendon complex [10]. A study of a 30-s tapotement massage (tapping) compared to the control group resulted in a decrease in the H/M ratio of 69% [11]. The study of skeletal muscle’s biomechanical shear elastic modulus [12] provides evidence that a 7-min massage reduces muscle stiffness. The authors reported a short-term effect after cessation and a quick return to baseline values after massage. A two-minute follow-up showed no more significant difference to the baseline measure.

A recent non-pharmacological approach to treat muscular spasticity is peripheral magnetic stimulation (rPMS). rPMS induces an excitation of the efferent motor nerve of the muscle, which initiates a muscle action potential, and thus a single pulse achieves a twitch contraction. The benefit of this stimulation is that it can be applied painlessly and can also target the muscle’s deep nerve structures. rPMS has already shown evidence in some clinical cases as a helpful method for the treatment of muscular disorders (for review, see [13]). rPMS is said to be effective in conjunction with conventional treatment for an immediate reduction of muscle hypertonia and a decrease in pain. The non-pharmacological treatment of muscular hypertension with rPMS has been used as an adjuvant of classical therapies [14] and may have considerable importance in clinical and rehabilitative settings as well as in the field of sports physiotherapy.

When rapid repetitive stimulators became available, research on lowering muscle hypertension with rPMS was conducted. Research suggests that peripheral magnetic stimulation holds promise as a non-invasive and effective treatment for muscle spasticity. An early study of multiple sclerosis patients [15] showed positive results with various stimulation protocols. Results showed that the stimulation frequency had a minor impact on the amount of H-reflex amplitude decrease, which was mostly determined by the coil’s location and intensity. Massive stimulation by recruiting sensory afferents may be able to affect the plasticity of the central nervous system in children with cerebral palsy [16]. They investigated the potential short- and long-term effects of five sessions of common peroneal and tibial nerve stimulation on the decrease of ankle plantar flexor muscle spasticity. They found a significant difference in spasticity from baseline after the third session, which increased until the fifth session. In another study [17], a completely different stimulation protocol consisting of a 1-s burst at 25 Hz followed by a 2-s pause was used. A total of 5000 pulses were administered, triggering 200 muscle contractions. Two weeks of this rPMS protocol applied twice daily to the paretic upper extremity did not improve motor function. Only short-term effects (after the first rPMS session) in the wrist flexors and long-term effects (after the 2-week intervention period) in the elbow extensors were observed in patients with mild to moderate spasticity as a result of rPMS.

The aim of our study was to compare the effectiveness of a passive muscular intervention (tapotement massage) with direct neuromuscular stimulation (rPMS). Repeated afferent input can influence (via activation of proprio-receptors) the spinal and cortical sensory circuitries [18,19]. Via repetitive peripheral magnetic stimulation and repetitive mechanical muscle-tendon stimulation, both treatments have positive effects on the condition of the treated muscles. In rPMS, changes in length and tension in the muscle are caused by the twitch contractions, which in turn cause corresponding afferents. In rTTM, which initially causes purely passive elongations, the rapid change in length triggers a tendon reflex, which leads to twitch contractions comparable to repetitive peripheral magnetic stimulation. These, in turn, produce sensory stimulation that achieves therapeutically positive adjustments in the neuromuscular apparatus. What both stimulation methods have in common is that they cause comparable afferents through changes in length and tension in the muscle. Our hypothesis is that we will find a comparable decrease in the reflex activity of the treated muscles, which will differ significantly from the sham group.

## 2. Materials and Methods

### 2.1. Participants and Experimental Design

Fifteen recreationally active university students were enrolled in the experiment, seven of whom were female and eight were male. They were on average 23.9 years old (±2.4 years), weighed 70.9 kg ± 12.7 kg, and stood 1.77 m tall ± 0.09; their body mass index was on average 22.1 ± 2.2. The volunteers did not show any abnormal, orthopedic, or neurological symptoms. All subjects were treated on the left leg, their non-dominant foot. No medication or central-nervous-system-active drugs (alcohol or nicotine) were consumed 24 h before the experiment. This study was performed in accordance with the Declaration of Helsinki and with the consent of the local ethics committee (Reg. No. A20160052) as required [20]. The study was carried out as a randomized control trial based on the CONSORT recommendations [21]. All subjects gave their written informed consent form before participating in the study.

**Figure 1:**
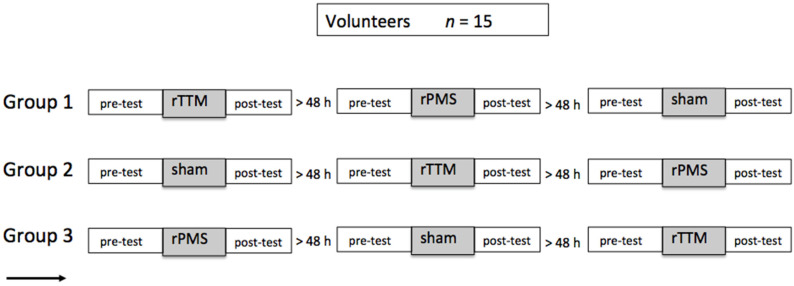
The measurement schema describes the course of the experiment. Participants were randomly assigned to a stimulation procedure in the three sequence conditions. A minimum period of 2 days was maintained between the different conditions (repetitive tendon tapotement massage = rTTM; sham = sham stimulation; rPMS = repetitive peripheral magnetic stimulation).

A pre- and post-test design was used to examine the effects of the 5 Hz repetitive peripheral magnetic stimulation and the 5 Hz Achilles tendon tapotement massage. In a third control condition, the participants were treated with 5 Hz sham rPMS stimulation; a 1% stimulator intensity was used to trigger a simulated acoustic output. The participants were divided into three groups (see Figure 1), which were randomly passed through the three different stimulation conditions. A minimum period of 48 h followed between the respective experimental interventions in order to exclude the influence of possible long-term effects. It was known that treatments exist that have longer-lasting effects under certain treatment conditions, but this was not the case in the present experiment (for reviews, see [22,23]). The stimulation was applied only once, and previous results showed hardly any effects two hours after administration [24]. Although we did not expect any series effects with an interval of 48 h between the stimulations, we scheduled three groups with different orders of stimulation. Measurements were taken immediately before and immediately after the intervention.

### 2.2. Data Recording and Processing

Signals were recorded and stored using signal processing software (DIAdem 8.1, National Instruments, Dublin, Ireland) for offline analysis. The recording occurred with a DAQ-Card 6024 (National Instruments, Austin, TX, USA) at a 12-bit resolution and with a sampling rate of 10,000/s. The data analysis was carried out using custom-programmed Visual Basic sequences in DIAdem.

### 2.3. Electromyography

The compound muscle action potential (CMAP) was measured as a peak-to-peak value to determine the T-reflex response. For that, electromyogram (EMG) signals were collected from Ag–AgCl cup electrodes (Hellige baby-electrodes; GE Medical Systems, Milwaukee, WI, USA) with a contact surface of 12 mm^2^. Electrode cream (GE Medical Systems Information Technologies, Milwaukee, WI, USA) and the adhesive rings (Hellige, General Electric Medical Systems Information Technologies, Milwaukee, WI, USA) were applied before starting the experiment. The spot on the muscle belly was determined visually and by palpation. Electrodes were attached to the soleus in an orientation that maximized their proximity to the presumed fiber path, with a distance between the centers of the measuring electrodes of 20 mm. If necessary, hair was removed from the relevant skin region, and the skin was cleaned using alcohol. Skin preparation reduces skin resistance, which is the prerequisite for better results in EMG measurements. To increase the hold and to ensure that the electrodes remained in place during the experiment, they were additionally fixed with a flexible tape (Fixomull Stretch, BSN medical, Hamburg, Germany). The electrode cables were twisted (decimation of antenna effect) and fixed with adhesive tape. The electrode position was marked with a permanent marker, and the application was documented by photographs so that it could be re-applied as precisely as possible. After attaching the electrodes, the skin impedance was measured at 30 Hz. In a few cases, if the impedance exceeded 10 kΩ, the sanding and gluing process had to be repeated.

EMG signals were amplified with a modified Biovision differential amplifier (Biovision, Wehrheim, Germany). The amplifier has a bandwidth of 1 Hz–1000 Hz, a × 1000 amplification, and an input impedance of 10 gΩ. A Butterworth 1st-order high-pass filter with a cut-off frequency of 1 Hz [25,26] was used to remove movement artifacts in the EMG.

### 2.4. Tendon Reflex Administration

The effects of the respective treatments and control condition (rPMS—rTTM—sham stimulation) were obtained by recording the tendon reflexes (TR). To exclude as many confounding factors as possible during the reflex measurement, some precautions were taken. A foil around the measuring stand shielded the test subjects from visual stimuli from the environment. Further, the test subjects wore noise-cancelling on-ear headphones (Bose QuietComfort 25), which filtered environmental noise, to protect against disruptive acoustic stimuli. In addition, brown noise (80 dB) was added through speakers behind the subjects to mask noise in the room as much as possible. The experimenters behaved as calmly and inactively as possible. During the measurements, test subjects were instructed to try to be relaxed, look straight ahead, and not speak or move. The latter was monitored by the investigator using a camera. Before the series of measurements began, the subjects were familiar with five test strokes on the tendon.

Reflex measurements were carried out on a test apparatus in which the subjects sat upright in a comfortable and reproducible position. The angles in the knees, hips, and feet were positioned at 90°. The adjustable components of the measuring stand (chair height, backrest, and position of the clamps) were individually adapted to each test person. Care was taken that the left leg was not clamped too tightly in the apparatus, as the reflexes could otherwise have been affected by cutaneous afferents. The left foot was fixed on a 32 °C warm plate with Velcro tape. At the rear end of the plate, there was a wooden device against which the calcaneus was placed.

To trigger a reflex, test subjects were given a short tap (12 ms) on the Achilles tendon. This was carried out with a small hammer, which was realized by the linear motor STA1104 with a Xenus servo controller type ACJ-090-12-S (Copley Controls, Canton, MA, USA). The acceleration path of the hammer was 60 mm, and the maximum speed was 5.3 m/s, to enable the shortest possible impact with high intensity. The push rod was accelerated to such an extent that the impact forces acting on the tendon were at least 40 N to trigger supra-maximal reflex responses [27]. Precise positioning of the foot was important so that the hammer could hit the Achilles tendon exactly in the middle, just above the lateral malleolus. Ten reflex responses were measured in both the pre- and post-tests of the tendon taps. There were always more than 10 s between the individual reflex releases to prevent post-activation depression. Before the start of the 10 measurements [28,29,30], five test strokes were made in order to ensure that the test subjects were familiar with the taps. Piezoelectric force sensors (Type 9011A, Kistler Instruments, Winterthur, Switzerland) have been mounted on the reflex hammer and the footplate to monitor the plantar pressure torque and the impact force at the Achilles tendon. The force-sensor data were prepared for the online presentation by using a Kistler charge amplifier type 5037A (Kistler, Winterthur, Switzerland).

### 2.5. Repetitive Peripheral Magnetic Stimulation Protocol

Repetitive peripheral magnetic stimulation (rPMS) was applied using a Magpro R100 stimulator (MagVenture, Skovlunde, Denmark). A butterfly coil type D-B80 (MagVenture, Skovlunde, Denmark) was used to achieve the most focal activation of the nerve with biphasic pulses of 280 μs duration. Participants are stimulated in an upright position to allow optimal accessibility of the coil to the nerve with the knee joint extended. The coil location was found by the systematic movement of the coil over the region of the tibial nerve in the popliteal fossa. The optimal position was found in that the lowest stimulation intensity at this position could still cause a palpable contraction of the calf muscles compared to neighboring stimulation sites [31]. The handle of the coil points vertically downward, with the current flow from the distal to the proximal direction over the nerve [32].

The experimenters who performed the stimulations were instructed in detail and experienced in the administration of the stimulation. In this experiment, no subject experienced any pain or other discomfort during rPMS. In order to optimally innervate the tibial nerve, the test subject stood hands-first against a wall, with the front right knee bent and the left leg extended backward (at an angle of 180°). The magnetic coil was placed in the upper area of the posterior crurius region, in the immediate vicinity of the tibial nerve. During the stimulation of the left tibialis nerve, contractions of the gastrocnemius and soleus muscles produced plantar flexion and relaxation of the calf muscle.

**Figure 2:**
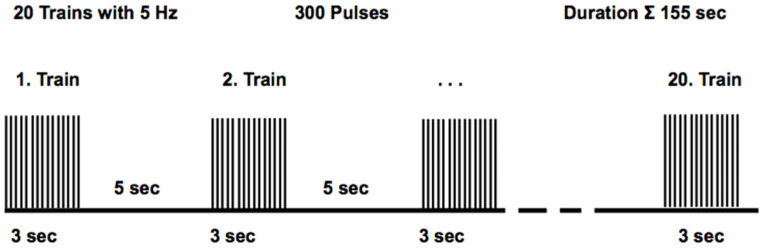
The protocol for repetitive peripheral magnetic stimulation (rPMS) on the tibial nerve and identically for mechanical stimulation of the Achilles tendon (rTTM) consisted of stimuli at a frequency of 5 Hz with 15 pulses over 3 s in one train. The breaks between trains lasted 5 s. A total of 20 trains with 300 pulses were administered over a period of 155 s.

The stimulation protocol [33] was delivered over 155 s in total in a 3 s on, 5 s off cycle (Figure 2). The stimulator generated pulses, with stimulation intensity at 94 A/μs (corresponding to 60% stimulator output), 5 Hz frequency, and 20 stimulation trains. A train consisted of 15 stimuli with 5 pulses per second (5 PPS) and an inter-stimuli interval (ISI) of 200 ms. Thus, the total number of applied pulses was 300. The coil did not overheat during a stimulation session as the range of stimulations was limited. During the sham stimulation, the same procedure (subject position, coil positioning, sham stimulation duration) was conducted, but the stimulation intensity was turned to 1% stimulator output, which could not excite nerves to depolarization but could trigger acoustic output.

### 2.6. Repetitive Tendon Tapotement Massage Protocol

**Figure 3:**
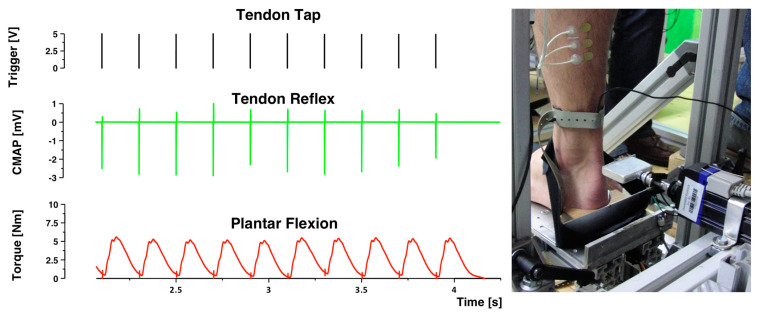
The impulses of the mechanical stimulation train triggered submaximal TR responses, which in turn resulted in moderate plantar flexion moments. Ten stimulations as a section of a three-second train were shown here on the left. Fifteen impulses were administered in one train. On the right side, the servo actuator, which applied an impact with a small hammer head to the Achilles tendon at the level of the lateral malleolus, could be seen. The foot of the volunteer was fixed with two Velcro straps to the footplate. Each subject was individually positioned on the impactor.

The repetitive tendon tapotement massage stimulation (rTTM) was administered via a servo actuator (Copley Controls, Canton, MA, USA). The actuator delivered mechanical stimuli to the Achilles tendon (Figure 3). The stimulation characteristics were 5 Hz frequency and 10-mm amplitude in the present study. This distinguishes the stimulation properties of this study from most other focal muscle-tendon vibration studies, which were performed at significantly lower amplitudes (≤1 mm) and considerably higher frequencies (between 80 Hz and 100 Hz). The mechanical muscle-tendon impacts were administered in a way that a potential tonic vibration reflex (TVR) could not have developed to impact the results [34]. Rigorous care was taken to ensure that no background EMG activities were detectable during the stimulation.

The stimulation carried out differed significantly in its physiological effects. The rTTM afferents are caused by passive elongation of the muscle-tendon complex. On the other hand, magnetic stimulations cause direct excitation of the efferent motor nerves and, as a result, afferents with different properties. The protocol of the tapotement massage in terms of duration, frequency, and intensity was comparable to the magnetic stimulation as described above, with the only difference that the tapotement stimulation took place in the sitting position. The stimulation protocol was exactly the same as with magnetic stimulation. Each impact of the actuator produced a tendon reflex and moderate plantar flexion torque. This, in turn, meant that the spinal circuitry received a strong passive mechanical input and an input from a following weak muscular twitch contraction. Brisk mechanical impacts generated by massage or vibration could affect the focally stimulated tissue [35]. In our experimental setup, we were unable to detect any signs of micro-traumatization of the stimulated tissue; there was no discomfort, irritation, or hematoma, which would have given us an indication of possible soft tissue injuries.

## 3. Data Analysis and Statistics

The reflex data were tested for the assumption of a normal distribution using the Shapiro–Wilk test. Levene’s test was used to assess the equality of variances for groups. The effect of stimulation on the T-reflex amplitude (the dependent variable) was analyzed using an analysis of variance with repeated measures (rANOVA) in a 3 (treatment) × 2 (time) design [36]. Changes within the treatment groups from pre- to post-test were tested for significance with single-sided paired *t*-tests. The decision to use the one-tailed test was based on the fact that both repetitive peripheral magnetic stimulation and tapotement massage therapies have been shown to improve the condition of the treated muscles.

A *p*-value of <0.05 was considered significant. Data obtained at the pre- and post-tests were given as the mean value ± standard deviation. The effect size was calculated for groups of equal size, and changes within groups between pre- and post-test were computed based on Cohen 1988 [37,38]. The effect-size partial eta-square (η_p_^2^) was considered with an η_p_^2^ > 0.01 as small, η_p_^2^ > 0.06 as medium, and η_p_^2^ > 0.14 as large, and the effect size d for significant results was considered with d > 0.20 as small, d > 0.50 as medium, and d > 0.80 as large. All data were calculated using SPSS version 29 (SPSS Inc., Chicago, IL, USA).

## 4. Results

The current experiment shows that both repetitive peripheral magnetic stimulation and tapotement massage via repetitive mechanical muscle-tendon stimulation had a significant reduction in muscular reflex activity after treatment compared to rPMS sham stimulation. The rANOVA showed that the changes over time resulted in a statistically significant interaction for treatment groups with F_(2,42)_ = 4.86, *p* = 0.013 *, and η_p_^2^ = 0.188 (see Table 1). The power was 1-β = 0.77, and the Shapiro–Wilk test indicated that all data were approximately normally distributed. Levene’s test of equality of error variances was not statistically significant with *p* = 0.739, and this met the assumption of homogeneity of variances for groups. The T-reflex-induced CMAP_pp_ (difference between pre- and post-test) was statistically different between treatment groups. In contrast to sham stimulation, both the rTTM group and the rPMS group showed a reduction in spinal excitability.

The mechanical stimulation rTTM achieved a decrease in CMAPs from 1.90 mV (±0.94) to 1.57 mV (±0.73). After the mechanical treatment, a single-sided paired *t*-test revealed a significant difference (*p* = 0.002, d = 0.87, 95% CI [0.26, 1.46]). As a result, there was a 17.4% reduction in reflex responses after the application of mechanical tapotement stimulation.

**Figure 4:**
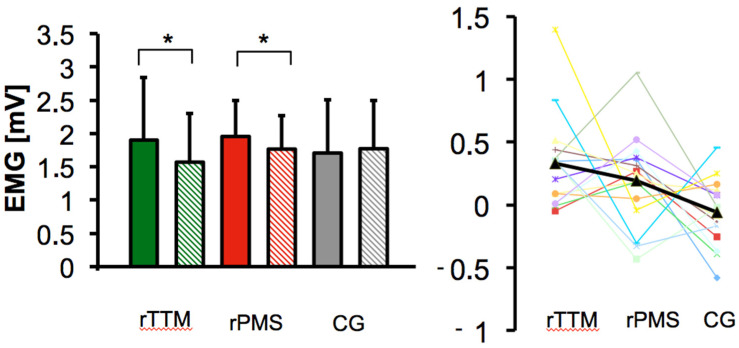
Changes in the compound muscle action potential (CMAP) of the soleus T-reflex amplitude following repetitive tendon tapotement massage (rTTM) of the Achilles tendon and repetitive peripheral magnetic stimulation (rPMS) on the tibial nerve compared to the control treatment (CG) with sham rPMS stimulation are demonstrated. Each color represents a treatment group; full color represents the pre-test, and hatched color represents the post-test. * Refers to the statistical significance comparison between the pre- and post-tests at the level of a 5% probability of error. The figure on the right shows the individual mean differences of the spinal reflex responses between conditions. The dark line represents the group mean values.

A reduction in CMAPs is also found within the rPMS group. The single-sided paired *t*-test indicated reduced peak-to-peak amplitudes of the treatment group (*p* = 0.034, d = 0.51, 95% CI [0.04, 1.04]) with a decrease from 1.96 mV (±0.54) to 1.77 mV (±0.51) by 9.8%. The mean values of the pre- and post-examinations of each treatment group are shown in Figure 4. In contrast to both rPMS and rTTM stimulations, there was no statistically significant change in the sham stimulation group. The raw data of the sham stimulation showed a mean value of 1.71 mV (±0.79) before and 1.77 mV (±0.73) after stimulation. The group mean values indicated a small increase of 3.38%. The single-sided paired *t*-test revealed no significant change in the soleus muscle response after the sham stimulation, with *p* = 0.210 in the control condition.

Comparing the mechanical rTTM with the rPMS, rTTM achieved a greater decrease in CMAPs. The rTTM had a reduction of the reflex response by 17.4% compared to the rPMS by 9.8%. The difference between the two treatments was 7.6%, but the paired *t*-test indicated no statistical significance (*p* = 0.40, d = 0.23, 95% CI [−0.29, 0.73]).

## 5. Discussion

The objective of this study was to evaluate changes in evoked muscle responses between two different types of sensory stimulation (tapotement massage and peripheral magnetic stimulation) of the muscle-tendon complex and sham stimulation. A decrease in reflex responses has already been recorded for the treatment with rPMS since the early studies of Nielsen et al. [15,39,40]. A decrease in the reflex amplitude is also demonstrated with low-frequency impacts from massage [8,9,11,12]. Our study focuses on which sort of low-frequency treatment is more efficient.

Muscle hypertonia or spasticity is associated with considerable, long-term stress on the patient and requires comprehensive therapy. This includes pharmacological as well as non-pharmacological interventions to reduce spasticity. One of the new non-pharmacological interventions is the use of repetitive peripheral magnetic stimulation. It has been reported previously that tendon jerks appear to be exaggerated due to the over-excitability of the stretch reflex in patients with spasticity [41,42,43,44]. However, it has been shown in spastic patients that a lack of inhibitory mechanisms is partially responsible for the uncoordinated contraction/relaxation of antagonistically working muscles [45,46]. The main difference between mechanical tapotement massage stimulation of the Achilles tendon and peripheral magnetic muscle stimulation is that the former results in passive elongation of the muscular tendon complex, whereas the latter leads to twitch contractions. These brisk repetitive stretches (5 Hz) of the tapotement massage in our study produce strong sensory muscle spindle afferents (Ia), which then excite the spinal alpha motor neurons supra-threshold [45,47,48] and trigger twitch contractions in the muscle. In contrast, the 5 Hz peripheral magnetic stimulation causes an excitation of the efferent motor nerve of the muscle’s motor neurons [49], causing it to generate a compound action potential and, as a result, a 5 Hz twitch contraction of the muscle.

### 5.1. Magnetic Stimulation

rPMS is a recently studied treatment that uses multiple, short-duration muscle stimulations to manipulate the neuro-muscular complex. rPMS is known as an adjuvant therapy that has been investigated several times and is specifically designed to reduce muscle hypertension [14,17,50], restore optimal muscle length, reduce pain [51], and improve muscular function [52,53]. rPMS may also be effective in conjunction with conventional treatments like self-myofascial release [54,55] for an immediate reduction of muscle hypertonia and a decrease in pain. An early study from our laboratory showed no statistically significant changes in H-reflex responses [56] after rPMS, which is most likely due to insufficient stimulation intensity, as [15] already pointed out the importance of stimulation intensity. A recent study [57] has shown that theta-burst stimulation, when applied directly to spastic muscles, can lead to a significant reduction in spasticity. They used 10 bursts (three stimuli at 50 Hz), repeated at a theta frequency of 5 Hz every 10 s, for a total duration of 200 s. This stimulation protocol is roughly comparable to the protocol we chose, in which the twitch contraction occurs at 5 Hz. Only the intensity of the theta-burst stimulation triggered by the triple pulse corresponds to stronger contractions. In this clinical study, a significant reduction of spasticity (Ashworth scale) and, accordingly, a reduction in Botox administration were achieved. This is in line with our findings that treatment with a 5 Hz rPMS consisting of 300 pulses over 155 s significantly reduces reflex responses in the soleus muscle by 9.8%. However, further studies will be needed to carry out a more thorough evaluation of the efficacy of the combination of different treatments.

### 5.2. Mechanical Stimulation

It is known that massage leads to a short-term reduction in muscle stiffness [12]. However, there is a significant difference between the effect of vibrations on the muscle-tendon complex, which is usually applied at higher frequencies, and the use of massage techniques, which by their very nature can only be used in the low-frequency range. Another significant mechanical difference between the impact of higher-frequency vibrations on the muscle-tendon complex and the different massage techniques is that the massage is applied with large amplitudes. With the high-frequency application, the vibrations can only apply small amplitudes to the muscle or tendon structure. These purely mechanical differences between the various treatments generate very different physiological effects since different sensory afferents are generated depending on the frequency and amplitude of the impact. In the case of the high-frequency vibration in the range of 75 Hz of the muscle-tendon complex, facilitating effects have been described [58]. By comparing the effects of interventional muscle tendon and cutaneous vibration, it is possible to evaluate the role of prolonged Ia-afferent activation in influencing cortico-spinal excitability changes [59]. Clear facilitation of the cortico-spinal excitability was observed when vibrating the tendon at 75 Hz and 120 Hz, with the strongest effect seen during 75 Hz vibration. In contrast, no distinct changes were seen when vibrating at 20 Hz.

Low-frequency mechanical tapotement massage of the Achilles tendon in our experiment differs from magnetic muscle stimulation in that it creates brisk and high-amplitude passive stretching of the muscle-tendon complex. Our experiment shows that there is, likewise, a significant reduction in reflex responses after treatment with a 5 Hz Achilles tendon tap with 300 pulses over a period of 155 s at the muscle. The decrease of 17.4% in reflex responses after the application of mechanical tapotement massage is obviously greater than with magnetic stimulation, which showed only a 9.8% reduction in reflex responses. The rTTM probably stimulates the entire muscle rather than parts of a muscle and stimulates muscles of the entire Achilles tendon complex at the same time, which may therefore result in quantitatively greater effects. Statistically, however, the two results do not differ significantly.

### 5.3. Limitations of the Study

We would like to point out that we examined healthy, uninjured young subjects. It cannot be ruled out that neurological disorders in patients will show different consequences of treatment since neurological disorders affect signal transmission and signal processing at the neuronal level, which can then have different effects compared to healthy subjects.

## 6. Conclusions

The present study demonstrates that the application of both tapotement massage (rTTM) applied at a 5 Hz stimulation rate and 5 Hz repetitive peripheral magnetic stimulation (rPMS) brings about a reduction in spinal neuromuscular excitability, as evidenced by a decrease in the amplitude of the T-reflex compared to sham stimulation. While massage appears to be more effective than magnetic stimulation, the effects were not significantly different from each other. These findings indicate that both repetitive peripheral magnetic stimulation (rPMS) and repetitive tendon tapotement massage (rTTM) can reduce spinal reflex excitability in healthy subjects. These observations have potential implications for the use of low-frequency afferent stimulation in the rehabilitation of both chronic and acute movement disorders. The present results suggest that rPMS and rTTM can be used to modulate muscle stiffness in a variety of contexts, such as rehabilitation, sports performance, and even daily activities. Furthermore, this research highlights the potential of rPMS and rTTM to be used as non-invasive interventions to improve muscle stiffness and reduce pain. Future studies should focus on determining the optimal dose and timing of repetitive peripheral magnetic stimulation (rPMS) and repetitive tendon tapotement massage (rTTM) for various clinical and performance goals.

## Figures and Tables

**Table 1:** A comparison of the raw data of the CMAP [mV] between the different treatments (rTTM, rPMS, and sham) is shown in the table. Significant differences in CMAP characteristic parameters are presented. * *p* < 0.05.

Group	CMAPpre	CMAPpost		Group Effect
			F	*p*-Value η_p_^2^ Partial
rTTM	1.90 ± 0.94	1.57 ± 0.73	4.86	* 0.013 0.188
rPMS	1.96 ± 0.54	1.77 ± 0.51		
sham	1.71 ± 0.79	1.77 ± 0.73		

## Data Availability

The data presented in this study are available on request from the corresponding author. Data are not publicly available due to access to the data being guaranteed by the first author as the data is not in ASCII code.
